# Bacterial diversity and Clostridia abundance decrease with increasing severity of necrotizing enterocolitis

**DOI:** 10.1186/s40168-015-0075-8

**Published:** 2015-03-23

**Authors:** Valarie E McMurtry, Raegan W Gupta, Lynn Tran, Eugene E Blanchard, Duna Penn, Christopher M Taylor, Michael J Ferris

**Affiliations:** Department of Microbiology, Immunology, and Parasitology, Louisiana State University Health Science Center, 1901 Perdido St., New Orleans, Louisiana USA; Department of Pediatrics, Children’s Hospital, 200 Henry Clay Ave., New Orleans, Louisiana USA; Pediatrix Medical Group of Louisiana, Baton Rouge General Medical Center, Baton Rouge, LA USA; The Research Institute for Children, 200 Henry Clay Ave., New Orleans, Louisiana USA

**Keywords:** Clostridia, Microbial diversity, Microbiota, Necrotizing enterocolitis

## Abstract

**Background:**

Necrotizing enterocolitis (NEC) is a devastating neonatal gastrointestinal disease that primarily affects premature infants. It is characterized by bowel inflammation and necrosis. In spite of extensive research, there has been little progress in decreasing the incidence or mortality of NEC over the past three decades. The exact etiology of NEC has not been identified. However, it is believed to result from an inappropriate immune response to gut microbiota. Using 454-pyrosequencing analyses of 16S rRNA genes that were PCR-amplified from stool DNA specimens, we compared the gut microbiota of infants with NEC to matched controls without NEC. The infants with NEC were then categorized into three subgroups based on severity: mild, severe, and lethal. We compared the microbiota among these subgroups and between each severity group and appropriate controls.

**Results:**

Bacterial diversity and the relative abundance of Actinobacteria and Clostridia were significantly lower in NEC specimens compared to controls. The absence of Clostridia was significantly associated with NEC. Microbial diversity and Clostridia abundance and prevalence decreased with increasing severity of NEC.

**Conclusions:**

Low bacterial diversity in stool specimens may be indicative of NEC and the severity of NEC. The low bacterial diversity, and the lack of Clostridia in lethal specimens, could indicate that the presence of a diverse bacterial population in the gut as well as the presence of taxa such as Clostridia may play a role in attenuating inflammation leading to NEC.

**Electronic supplementary material:**

The online version of this article (doi:10.1186/s40168-015-0075-8) contains supplementary material, which is available to authorized users.

## Background

Necrotizing enterocolitis (NEC) is a severe neonatal inflammatory bowel disease that primarily affects premature infants [1]. It can lead to bowel necrosis requiring surgical removal of the affected tissue. Mortality and morbidity are high: up to 50% of cases that require surgery result in death [2]. Depending on location and extent, surgical bowel removal may result in prolonged dependency on parenteral nutrition and vitamin administration, slow growth rate, neurodevelopmental delays, and possibly a bowel transplant [1,3]. Infants with NEC typically have longer and more costly hospital stays, and the increased healthcare costs continue through the first 3 years of life for survivors of surgically treated NEC [4,5]. Unfortunately, there has been no decrease in either the incidence or the mortality rate of NEC over the last three decades, indicating a need for better treatment options and improved preventative measures [6].

The exact etiology of NEC is not known and presumed to be multifactorial. While evidence suggests that the presence of bacteria in the gut is necessary for pathogenesis [7,8], a combination of inappropriate immune responses, genetic predisposition, microvascular tone, and bacteria in the gut all may contribute to the disease [9]. There is no consensus regarding the diversity or composition of microbiota associated with this disease. For example, two studies show that diversity is significantly decreased in NEC, both at diagnosis [10] and in the first 2 weeks of life [11], while three others show no difference in diversity between NEC and controls in the week prior to NEC or within 72 h of diagnosis [12-14]. Concerning the relationship between the composition of microbial communities and NEC, some investigations show that Proteobacteria are increased prior to and during the onset of NEC [11,12,14], and others demonstrate that Enterobacteriaceae, a family within the phylum Proteobacteria, are decreased in infants with NEC within the week prior to diagnosis [15]. A predominance of both Firmicutes and Proteobacteria has been hypothesized to be related to the time of onset of NEC with a predominance of Firmicutes during day of life (DOL) 4 to 7 having an earlier onset than Proteobacteria predominance during DOL 10 to 16 [11]. Finally, some studies show no difference in bacterial composition between NEC and controls [13,16]. Thus, no individual species or bacterial community has been found to be consistently associated with NEC.

Although it is well recognized that not all cases of NEC are equally severe, the level of severity is not typically a variable considered in analyses of the microbiota of patients with NEC, even though there are clinical differences in severity and resulting treatment [17]. We postulated that the microbiota of infants with the most severe form of NEC, that is, those that succumbed to the disease, would be significantly different from that of controls. Conversely, the microbiota of infants with less severe forms of NEC might more closely resemble that of controls. We stratified NEC cases into three categories, mild, severe, and lethal, based on severity and outcome, and compared the microbiota across each category as well as matched controls for each group. We also compared the microbiota of all NEC cases to those of matched controls.

## Results

### Patient cohort

Based on the 12 clinical parameters evaluated, our cohort of 21 NEC infants was well-matched with the 74 controls (Additional file 1: Table S1). Considering the total cohort, NEC subjects tended to be slightly less mature than their control counterparts and tended to have more frequent use of H2 blockers. There were occasional trends within the subgroups, as well as a significant difference in caffeine administration between NEC and control subjects in the mild NEC group. However, this finding was not duplicated in the other subgroups nor in the total cohort and may simply reflect the small numbers. It should be noted that we excluded antibiotics given on the day of diagnosis from the definition of recent antibiotic administration. Many of the specimens collected from premature infants with NEC were taken on the same day as diagnosis. Thus, collection of the stool coincided with the start of intravenous antibiotics. However, it is unlikely that a single dose of antibiotics given within a few hours of collection would significantly change the bacterial genetic material in stool.

### Microbiota analyses

**Figure 1:**
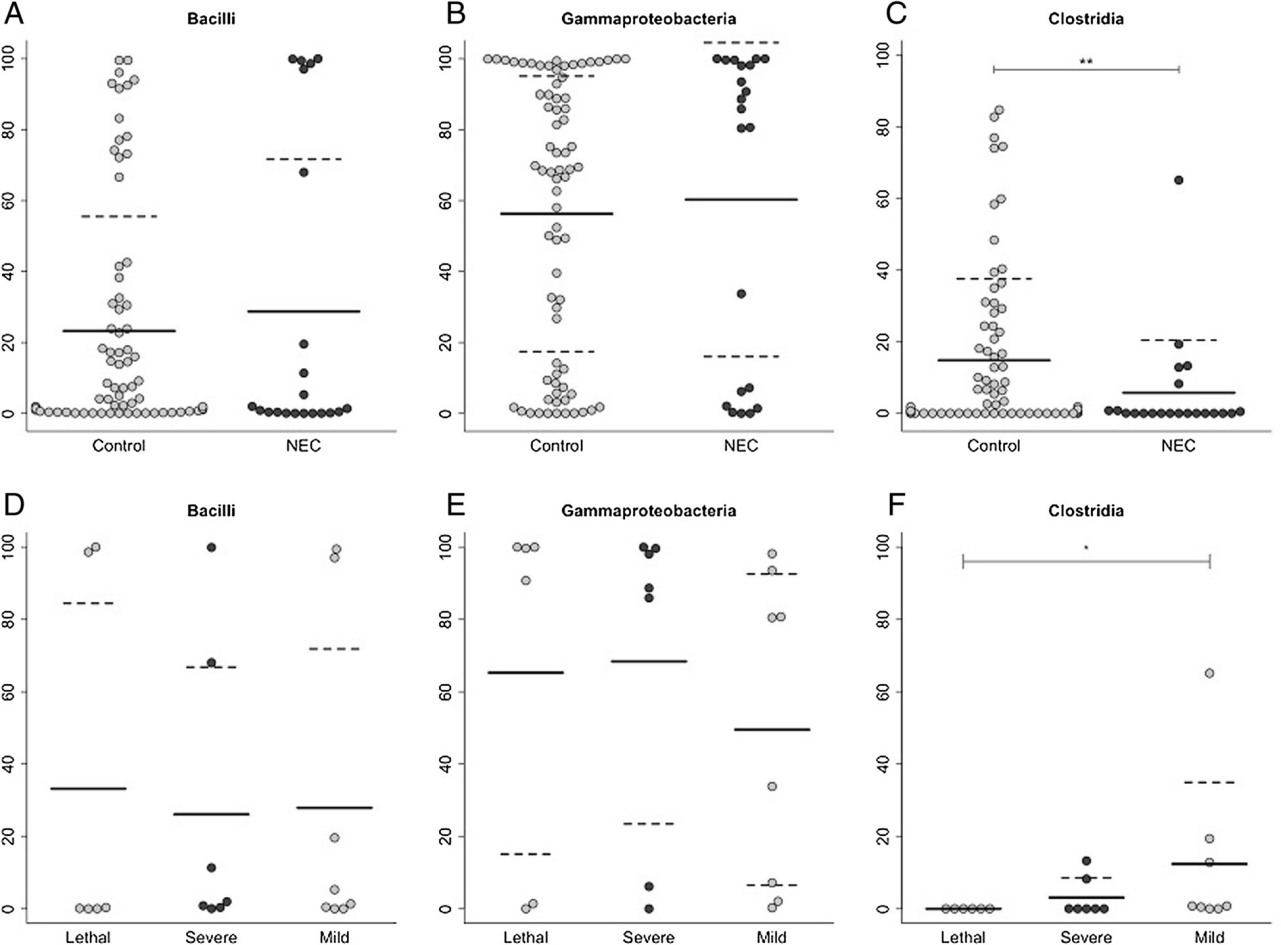
**Relative abundance of taxonomic differences between NEC stool specimens and controls.** Relative abundance of the most abundant taxa at class level **(A-C)** for NEC specimens (*n* = 21) compared to controls (*n* = 74) as well as the abundance at the different severities of NEC **(D-F)**. Clostridia was significantly reduced in NEC patients **(C)**, and as the severity of NEC decreased, the abundance of Clostridia increased. Lethal NEC, *n* = 6; severe NEC, *n* = 7; and mild NEC, *n* = 8. The solid line represents the mean, and the standard deviation is represented by dashed lines when between 0 and 100. **(A-C)** Significance was determined using a Mann-Whitney *U* test. For comparison of the NEC levels, Kruskal-Wallis test was used from **(D-F)**. *P value < 0.05, **P value < 0.01. NEC, necrotizing enterocolitis.

**Figure 2:**
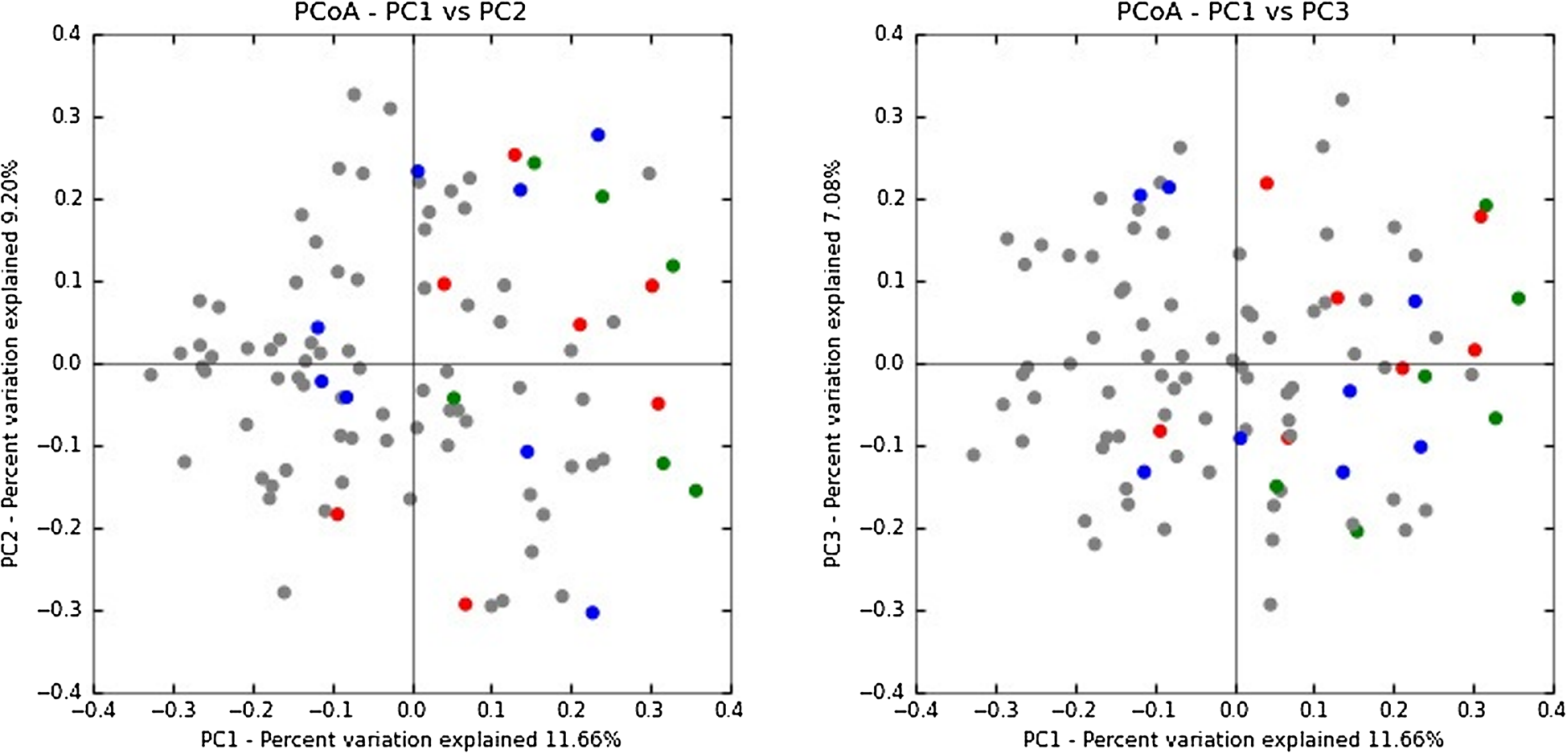
**Principal coordinate analysis (PCoA) of NEC compared to controls.** Unweighted UniFrac uses phylogenetic information to compare specimens that can be visualized with PCoA plots. The gray circles represent the control specimens (*n* = 74), blue circles represent mild NEC specimens (*n* = 8), red circles represent severe NEC specimens (*n* = 7), and green circles represent lethal NEC specimens (*n* = 6). ANOSIM was used to evaluate the UniFrac distances of all NEC *vs* controls. Although the *P* value is statistically significant (*P* = 0.001), the *R* value (0.2309) suggests that the communities are essentially not separable. PC, principal coordinate.

**Figure 3:**
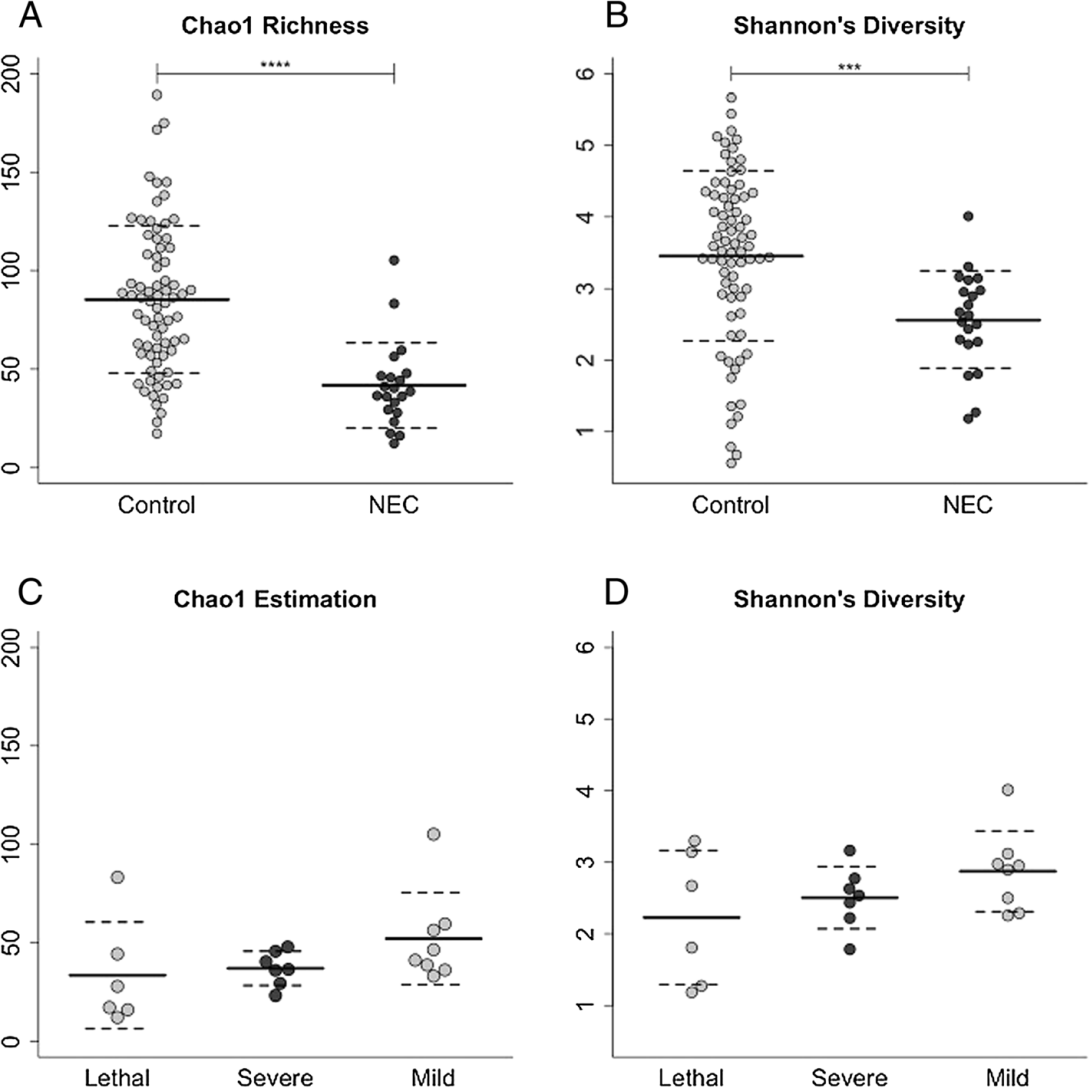
**Microbial alpha diversity.** Chao1 richness estimation **(A)** and Shannon’s diversity index **(B)** were used to measure alpha diversity. NEC specimens (*n* = 21) were significantly lower than controls (*n* = 74) for both measures of diversity. These measures were also used to compare the different severities of NEC **(C, D)**. Lethal NEC, *n* = 6; severe NEC, *n* = 7; and mild NEC, *n* = 8. Solid lines represent the mean with standard deviation represented by dashed lines. **(A, B)** were evaluated with a Mann-Whitney *U* test, and **(C, D)** were evaluated with a Kruskal-Wallis test. ***P value <0.001, ****P value < 0.0001. NEC, necrotizing enterocolitis.

Several notable differences were observed between the microbiota of total NEC specimens and controls and between the microbiota in the three categories of NEC. The results of all comparisons are summarized in tabular form (Additional file 1: Table S2). We found that the abundance of bacteria within the class Actinobacteria (Additional file 1: Table S2) and the class Clostridia (Figure 1C) was significantly lower in total NEC specimens compared to controls, *P* = 0.009 and *P* = 0.004, respectively. We found no difference in abundance of Bacilli (Figure 1A) or Gammaproteobacteria (Figure 1B). The abundances of two bacterial genera, *Veillonella* and *Streptococcus*, were significantly lower in total NEC specimens compared to controls, *P* = 0.007 and *P* = 0.002, respectively (Additional file 1: Table S2). Principle coordinate analysis of unweighted UniFrac distances showed no distinct separation between microbial communities in total NEC specimens and control specimens indicated by *R* values close to zero (Figure 2). Although the unweighted UniFrac *P* value is statistically significant (*P* = 0.001; *R* = 0.2309), the low *R* value suggests that the communities are essentially not separable, that is, NEC specimens are as different from each other as they are from controls. We found that both Chao1 richness (Figure 3A) and Shannon’s diversity (Figure 3B) were significantly lower in total NEC specimens compared to controls, *P* < 0.0001 and *P* = 0.0002, respectively. We performed an ANOVA using the operational taxonomic unit (OTU) abundance table to test whether there were any OTUs that were significantly different between NEC and controls using OTUs present in at least 25% of specimens (102 total OTUs). None were found to be significantly different after false discovery rate (FDR) correction (Additional file 1: Table S3).

We focused our analyses of the microbiota in mild, severe, and lethal categories of NEC specimens on diversity and class-level assessments of the microbiota. Lethal NEC specimens showed a complete absence of bacteria in the class Clostridia, and this class was significantly less abundant (*P* = 0.007) in these specimens compared to controls (Additional file 1: Table S2). The class Actinobacteria was significantly reduced in severe NEC specimens compared to controls, *P* = 0.044 (Additional file 1: Table S2). Chao1 richness, an indirect measure of richness, was significantly lower in all three categories of NEC specimens, lethal (*P* = 0.005), severe (*P* = 0.002), and mild (*P* = 0.003), compared to their respective controls (Additional file 1: Figure S1 A, C, E). Shannon’s diversity, an indirect measure of evenness, was also significantly lower in each category of NEC specimens compared to their respective controls, lethal *P* = 0.04, severe *P* = 0.03, and mild *P* = 0.01 (Additional file 1: Figure S1 B, D, F).

To investigate whether the prevalence, relative abundance, or diversity of specific bacterial taxa is associated with the degree of NEC severity, we compared the microbiota across the three categories of NEC. We found that as the severity of NEC increased, the prevalence and relative abundance of Clostridia decreased. In contrast, bacteria in the classes Bacilli and Gammaproteobacteria showed no similar trend (Figure 1D, E). The average abundance of Clostridia was 12% in mild NEC, 3% in severe NEC, and 0% in lethal NEC (Figure 1F), and Clostridia abundance in lethal NEC specimens was significantly lower than that in mild NEC specimens (*P* = 0.025). The average prevalence of Clostridia likewise decreased, 75% (6/8) in mild NEC, 43% (3/7) in severe NEC, and 0% (0/6) in lethal NEC. In light of this information, we used Fisher’s exact test to determine whether there was an association between the absence of Clostridia and the presence of NEC. Of the 74 controls, only 14 (19%) lacked Clostridia, while 12 of 21 (57%) of NEC specimens lacked this taxon (*P* = 0.002). The average Chao1 diversity and Shannon’s diversity also increased as the severity of NEC decreased (Figure 3C, D).

## Discussion

Studies of the microbiota of infants with NEC have revealed no consistent pattern of bacterial taxa associated with this disease. Since all NEC cases differ clinically, we stratified our NEC subjects into three categories based on the level of severity of the disease, postulating that the most extreme cases, those in which an infant succumbs to the disease, would provide the best opportunity to identify aberrations of microbiota statistically associated with NEC.

There are two findings of interest in this cross-sectional, case-control study of fecal microbiota in premature infants with and without NEC that suggest that variation in disease severity is correlated with a spectrum of bacterial dysbiosis. Bacterial diversity, as measured by both Chao1 richness and Shannon’s index, was clearly decreased in the NEC specimens compared to controls in the total NEC group (Figure 3), and this trend toward lower diversity was also observed across NEC subgroup; as diversity decreased, the level of NEC severity increased (Additional file 1: Figure S1). The level of microbial diversity in NEC is controversial. Some studies have found no difference in diversity between NEC and control samples a week prior or >72 h prior to diagnosis [12-14]. Our data supports the results of two investigations that found low bacterial diversity associated with NEC [10,11]. Furthermore, in our study, microbial diversity tended to decrease with increasing disease severity (Figure 3), supporting the concept of a spectrum of bacterial dysbiosis. In adults, low bacterial diversity in the gut has been associated with inflammatory bowel disease (Crohn’s disease) and with greater abdominal discomfort levels in patients with food intolerances [18,19]. In infants, low bacterial diversity during the first month of life preceded the development of asthma at school age [20]. These observations suggest that low intestinal bacterial diversity may negatively affect the immune system.

Regarding bacterial composition of the intestinal microbiota, Bacilli and Gammaproteobacteria accounted for most of the relative abundance in both control and NEC samples (Additional file 1: Table S2). Although the relative abundances of both classes varied widely across the control samples, they appeared to be clustered at abundance extremes in the NEC samples, again reflecting decreased diversity (Figure 1). This clustering phenomenon has been reported by other investigators [11] and belies the hypothesis that NEC is predominantly a Gram-negative disease.

A significant factor complicating any study of the microbiota associated with NEC is the almost universal administration of antibiotics to premature infants. Increased *Enterobacter* and decreased diversity have been found in premature infants who have received antibiotics early in life [21]. However, in our study, there was no difference between NEC and control groups’ past use of antibiotics (Additional file 1: Table S1), and *Enterobacter* abundances did not differ between NEC and controls (data not shown). It is unlikely that the first stool after administration of the first dose of antibiotics would show the changes present in following stool specimens since the composition of DNA from the microbiota would be the same as before the administration of antibiotics. At this time, it is unclear what factors predispose a microbiota to shift in one direction or another, but further research in this area may provide clues for preventive and/or therapeutic measures. Another limiting factor of this study is the use of specimens taken immediately prior to or shortly after the diagnosis of NEC. NEC is certainly a multifactorial disease, and other physiological processes that take place during NEC could affect the microbiota.

Although we found no bacterial taxon or community that was consistently associated with NEC, there appeared to be at least one notable *absence*. All six stool specimens from the infants with lethal NEC lacked representatives of the class Clostridia. In contrast, Clostridia contributed a mean relative abundance of almost 15% (range: 0% to 85%) in controls (Figure 1C). The percent abundance of Clostridia decreased as the severity of NEC increased (Figure 1F). Considering the entire infant cohort, the absence of Clostridia was strongly associated with NEC (*P* = 0.002, Fisher’s exact test). This is in agreement with the findings of Smith *et al*. who found *Clostridium* via *in situ* hybridization (FISH) in only 4 out of 24 surgical specimens from patients with NEC [22] corresponding to our severe NEC group.

Certain Clostridia, namely members of clusters XIVa, IV, and XVIII, have been shown to decrease inflammation in the gut during allergic diarrhea in mice and increase differentiation of FOXP3+ regulatory T cells (Tregs) [23]. Tregs play a vital role in gut immune homeostasis by inhibiting innate and adaptive immune responses [24-26]. Decreased Treg number and function predisposes the gut to increased inflammation. Weitkamp *et al*. reported that the proportion of Tregs, as well as ratios of Treg/CD4 T cells and Treg/CD8 T cells, were lower in lamina propria mononuclear cells isolated from surgical specimens of ileum from NEC patients compared with controls [27]. In a rat pup model, Tregs were reduced eightfold after induction of experimental NEC [28]. Administration of exogenous Tregs prior to induction of NEC resulted in reduced incidence of NEC, improved survival, and decreased tissue damage. The administered Tregs were shown to regulate dendritic cells by suppressing upregulation of CD80 and to modulate trafficking of effector cells in the mesenteric lymph nodes [28]. Decreased abundance of Clostridia has been linked to other examples of human inflammatory bowel disease, for example, Crohn’s disease [18,29,30], and to increased abdominal discomfort in cases of food allergies [19]. Whether the relative abundance of Clostridia in stool specimens may serve as a useful marker of the trajectory of gut microbial development toward health or disease warrants further investigation.

## Conclusions

In summary, a number of studies have focused on describing the composition of the gut microbiota associated with NEC [10,12-16]. However, there is still no consensus regarding the composition of gut bacteria associated with this condition. Rather than identifying the presence of certain bacteria in association with NEC, our data suggest that the *absence* of certain bacterial classes, such as Clostridia, and a reduction in bacterial diversity of the gut may represent risk factors for NEC. We postulate that the presence of these taxa and their immunoregulatory functions are necessary to prevent an overly robust inflammatory response. In the absence of these ‘protective’ taxa, the presence of a variety of other bacterial taxa can lead to exaggerated inflammation and NEC.

## Methods

### Ethical statement

This study is part of an ongoing, multicenter investigation of the factors affecting the postnatal development of fecal microbiota in premature infants. It was approved by the institutional review boards of Louisiana State University Health Sciences Center, Touro Infirmary, East Jefferson General Hospital, and Children’s Hospital of New Orleans (IRB #6728).

### Study population

Patients were continually enrolled in a study of the gut microbiota from 2007 to 2011. Written consent was obtained upon the patient’s admittance to the neonatal intensive care unit (NICU), and the patient’s stool was collected at each bowel moment until the patient was discharged from NICU. We choose 95 premature infants ≤1,500 g and/or ≤34 weeks of gestation that were enrolled in the study, including 21 infants with NEC and 74 age-, gestational age-, and birth weight-matched infants without NEC (controls). Other clinical parameters of the infants were noted, but not considered for matching (Additional file 1: Table S1). NEC was defined clinically by the presence of abdominal distension, feeding intolerance, bloody stools, radiographically by demonstration of *pneumatosis intestinalis* on abdominal X-ray (Bell stage II or greater) [17], and/or by pathology evidence of mucosal inflammation/obliteration and ischemia/hemorrhagic necrosis. Two of the 21 NEC patients did not exhibit *pneumatosis intestinalis* on abdominal X-ray. In each case, the diagnosis was suspected by clinical presentation and confirmed by pathological examination of surgical or autopsy specimens [31]. Surgically treated and lethal NEC cases were confirmed by pathology examination of surgical or autopsy specimens. For the purposes of our study, the premature infants with NEC were subdivided into three categories: mild, severe, and lethal. The mild NEC group (*n* = 8) included patients that were successfully managed medically by 10 to 14 days of bowel rest, decompression, and broad-spectrum intravenous antibiotics. The severe NEC group (*n* = 7) included patients that required prolonged medical management (>14 days) or surgical intervention due to intestinal perforation. The lethal NEC group (*n* = 6) included fatal cases, regardless of treatment. Stools from NEC infants were matched with three to five stools from control infants matched for gestational age (GA), birth weight (BW), and DOL when the sample was taken.

### Stool sample collection and analytic methods

One stool sample was included from each study subject. Stool specimens from patients with NEC were collected from 1 to 5 days prior to onset of symptoms or on the day of diagnosis. Control specimens (*n* = 74) were selected on the basis of age, gestational age, and birth weight from the pool of samples collected serially from infants enrolled in the multicenter study during their stay in the NICU. All samples were placed in a nucleic acid preservative at the time of collection and stored frozen until extraction of DNA using a Qiagen QIAamp mini kit (Qiagen, Venlo, The Netherlands), as previously described [32]. The microbiota in stool specimens was characterized using 454-pyrosequencing analysis of PCR-amplified segments of the V1 to V3 region of the 16S rRNA gene using primers 27 F: GAGTTTGATCNTGGCTCAG and 519R: GWNTTACNGCGGCKGCTG. Our forward primer is very similar to the 27 F bacterial primer that has been used in other studies of NEC [12,14]. The C at position 11 in the 27 F primer has been replaced by a mixture of A, T, G, and C; this allowed us to expand the number of bacterial taxa targeted by the primer. This includes an increase in the phyla Actinobacteria (23450 to 34037), Bacteroidetes (40349 to 55791), and Proteobacteria (113839 to 176807). The primer set used has a known bias against Gardnerella species, targeting 17/101 using the Ribosomal Database Project (RDP) probe match. The 519 reverse primer was used in order to target as many variable regions (V1 to V3) as possible using the 454-pyrosequencing platform. Sequences were analyzed using the QIIME pipeline [33]. A total of 657,504 sequences were included in the study. The average number of sequences per specimen was 6,921 with a minimum of 1,006 and a maximum of 25,980. The average sequence length was 355 bases. Sequences with >6 ambiguous bases, homopolymers >6 bases in length, any mismatches in the primer, or a mean quality score <25 on a scale of 0 to 40 were eliminated. Chimeric sequences were identified and removed using UCHIME [34]. Sequences were aligned using PyNast [35] and the Greengenes core reference alignment [36] and clustered into OTUs at 97% similarity using USEARCH [37] with a minimum of two sequences per OTU. A total of 1,277 OTUs were formed. A phylogenetic tree was created using FastTree [38]. Taxonomic identification of OTUs was performed using the RDP classifier using a confidence of 0.8 [39]. Bacterial diversity, as well as the prevalence and abundance of each phylum, class, and genus in each stool specimens, was calculated. We compared the prevalence and relative abundance of microbiota in stool specimens, at the phylum, class, and genus levels, as well as the diversity of OTUs, in NEC and control infants, and in NEC infants at each of three levels of severity at the phylum and class level. Diversity was measured by Chao1 and Shannon’s indexes using the average of 50 iterations in which 800 sequences were randomly subsampled from each specimen. Lethal NEC compared to matched controls, severe NEC compared to matched controls, and mild NEC compared to matched controls used data from the complete analysis instead of separate analysis to remove possible complicating factors that can result from clustering methods and randomization of subsampling in diversity estimations.

### Statistical methods

Continuous clinical variables were analyzed using the unpaired *t*-test. Discrete variables were analyzed using Fisher’s exact test with Yate’s continuity correction or, in the case of feeding type, Pearson’s chi-square test for independence (Instat 3 for Windows, GraphPad Software, La Jolla, California, USA, www.graphpad.com). The Kruskal-Wallis rank sum test was used to evaluate differences in the relative percent abundance of taxa or diversity in three or more categories of specimens followed by Dunn’s test of multiple comparisons (R version 3.1.1, stats and Dunn’s test). A Mann-Whitney *U* test was used to assess statistical differences in relative percent abundance of taxa in two categories of specimens (R version 3.1.1, stats). Weighted and unweighted UniFrac measurements were used to measure distances between microbial communities in two categories of specimens, and principle coordinate analyses were used to assess the separation between two categories of specimens [40]. Analysis of similarity (ANOSIM) in QIIME was used to evaluate the significance of unweighted and weighted UniFrac distances between categories [33]. Analysis of variance (ANOVA) with FDR correction was used within QIIME to determine whether OTU relative abundance is different between NEC and controls [33]. The acceptable type 1 error was set at *α* < 0.050 (two-tailed test).

## Availability of supporting data

The data set supporting the results of this article is included within the additional files as supplemental information. The sequences, quality scores, and specimen descriptions are publicly available at http://dx.doi.org/10.6084/m9.figshare.1327660.

## Additional file

Additional file 1:
**Data set supporting the results of the article. Table S1.** Clinical description of subject groups at time of specimen collection. **Table S2.** Differences in relative abundance. **Table S3.** ANOVA of OTU abundances. **Figure S1.** Diversity of lethal, severe, and mild NEC specimens compared to controls.

## Abbreviations

FDR: false discovery rate
NEC: necrotizing enterocolitis
NICU: neonatal intensive care unit
OTU: operational taxonomic unit
Tregs: FOXP3+ regulatory T cells
