# Supplementary material for: Bacterial diversity and Clostridia abundance decrease with increasing severity of necrotizing enterocolitis
Source: Microbiome. 2015 Mar 23;3:11. doi: 10.1186/s40168-015-0075-8 (PMC4373520; doi:10.1186/s40168-015-0075-8)
Supplement: Additional file 1: — Data set supporting the results of the article. Table S1. Clinical description of subject groups at time of specimen collection. Table S2. Differences in relative abundance. Table S3. ANOVA of OTU abundances. Figure S1. Diversity of lethal, severe, and mild NEC specimens compared to controls. [file 40168_2015_75_MOESM1_ESM.doc]

**Table S1:** **Clinical description of subject groups at time of specimen collection.**

Continuous variables were expressed as mean ± standard deviation. Total NEC: all cases of necrotizing enterocolitis; Lethal NEC: fatal cases of necrotizing enterocolitis; Severe NEC: cases of necrotizing enterocolitis that required surgical intervention or unusually prolonged medical management; Mild NEC: cases of necrotizing enterocolitis that responded to conventional medical management; GA: gestational age; BW: birth weight; DOL: day of life; M/F: male/female ratio; SGA/AGA: small for gestational age/appropriate for gestational age (1); STER+/STER-: presence or absence of maternal steroids, ABX+/ABX-: presence or absence of antibiotic administration within 3 days prior to, but excluding day of specimen collection; ABX Hx +/ABX HX-: presence or absence of distant history of antibiotic administration; VD/CS: vaginal delivery/Caesarean section; BM/F/M: feeding type (**B**reast **M**ilk/**F**ormula/**M**ixture of breast milk and formula); CAFF+/CAFF-: presence or absence of caffeine administration; H2+/H2-: presence or absence of Histamine-2 receptor blocker administration. Continuous variables were normally distributed and statistically analyzed using the unpaired t-test. Discrete variables were analyzed using the Fisher’s exact test with Yate’s continuity correction or in the case of feeding type, the Pearson’s Chi-squared test for Independence. The acceptable type 1 error was set at α < 0.050 (2-tailed test).

| **Parameter** | **Total NEC (n=21)** | **Controls (n=74)** | ***P-value*** |
| --- | --- | --- | --- |
| GA (weeks) | 27.2 ± 2.8 | 28.3 ± 2.5 | 0.083 |
| BW (grams) | 1037 ± 397 | 1111 ± 370 | 0.428 |
| DOL (days) | 26.7 ± 14.9 | 27.6 ± 14.5 | 0.805 |
| M/F | 11/10 | 38/36 | 1.000 |
| SGA/AGA | 2/19 | 9/65 | 1.000 |
| STER+/STER- | 14/7 | 42/32 | 0.461 |
| ABX+/ABX- | 2/19 | 1/73 | 0.122 |
| ABX Hx+/ABX Hx- | 17/4 | 63/11 | 0.736 |
| VD/CS | 9/12 | 18/56 | 0.108 |
| BM/F/M | 7/5/9 | 17/22/35 | 0.617 |
| CAFF+/CAFF - | 14/7 | 59/15 | 0.245 |
| H2+/H2- | 3/18 | 2/72 | 0.070 |

| **Parameter** | **Lethal NEC (n=6)** | **Controls (n=21)** | ***P-value*** |
| --- | --- | --- | --- |
| GA (weeks) | 27.4 ± 2.7 | 28.3 ± 2.3 | 0.413 |
| BW (grams) | 945 ± 404 | 1100 ± 353 | 0.366 |
| DOL (days) | 23.0 ± 14.2 | 23.1 ± 12.2 | 0.981 |
| M/F | 4/2 | 12/9 | 1.000 |
| SGA/AGA | 2/4 | 2/19 | 0.204 |
| STER+/STER- | 4/2 | 13/8 | 1.000 |
| ABX+/ABX- | 1/5 | 0/21 | 0.222 |
| ABX Hx+/ABX Hx- | 4/2 | 19/2 | 0.204 |
| VD/CS | 3/3 | 6/15 | 0.367 |
| BM/F/M | 1/3/2 | 5/5/11 | 0.463 |
| CAFF+/CAFF - | 5/1 | 17/4 | 1.000 |
| H2+/H2- | 1/5 | 0/21 | 0.222 |

| **Parameter** | **Severe NEC (n=7)** | **Controls (n=22)** | ***P-value*** |
| --- | --- | --- | --- |
| GA (weeks) | 25.1 ± 1.6 | 26.7 ± 2.2 | 0.082 |
| BW (grams) | 836 ± 241 | 893 ± 251 | 0.606 |
| DOL (day) | 28.6 ± 13.9 | 29.3 ± 14.6 | 0.906 |
| M/F | 6/1 | 10/12 | 0.093 |
| SGA/AGA | 0/7 | 3/19 | 0.558 |
| STER+/STER- | 4/3 | 12/10 | 1.000 |
| ABX+/ABX- | 1/6 | 0/22 | 0.241 |
| ABX Hx+/ABX Hx- | 6/1 | 20/2 | 1.000 |
| VD/CS | 3/4 | 6/16 | 0.642 |
| BM/F/M | 2/1/4 | 5/7/10 | 0.665 |
| CAFF+/CAFF - | 7/0 | 21/1 | 1.000 |
| H2+/H2- | 1/6 | 1/21 | 0.431 |

| **Parameter** | **Mild NEC (n=8)** | **Controls (n=31)** | ***P-value*** |
| --- | --- | --- | --- |
| GA (weeks) | 28.8 ± 2.8 | 29.3 ± 2.2 | 0.656 |
| BW (grams) | 1281 ± 411 | 1279 ± 389 | 0.989 |
| DOL (days) | 27.9 ± 17.6 | 27.8 ± 17.1 | 0.988 |
| M/F | 1/7 | 16/15 | 0.106 |
| SGA/AGA | 0/8 | 4/27 | 0.563 |
| STER+/STER- | 6/2 | 17/14 | 0.432 |
| ABX+/ABX- | 0/8 | 1/30 | 1.000 |
| ABX Hx+/ABX Hx- | 7/1 | 24/7 | 1.000 |
| VD/CS | 3/5 | 6/25 | 0.355 |
| BM/F/M | 4/1/3 | 7/10/14 | 0.264 |
| CAFF+/CAFF - | 2/6 | 21/10 | **0.045*** |
| H2+/H2- | 1/7 | 1/30 | 0.373 |

*Statistically significant at p<0.050.

**Table S2: Differences in Relative Abundance**: The mean relative percent abundance of the most abundant bacteria in the specimens was calculated at various taxonomic levels in NEC specimens from the three severity groups and compared to those of controls using a Mann-Whitney U-test. Phyla are bolded, Class level taxa are indented under the phylum, and genera are italicized and indented.

| **Taxon** | **Total NEC (n=21)** | **Control (n=74)** | **P value** |
| --- | --- | --- | --- |
| **Actinobacteria** | 1.29 | 1.71 | 0.024* |
| Actinobacteria | 1.24 | 1.67 | 0.009* |
| **Proteobacteria** | 60.88 | 56.86 | 0.407 |
| Gammaproteobacteria | 60.29 | 56.28 | 0.443 |
| *Klebsiella* | 5.09 | 3.88 | 0.110 |
| **Firmicutes** | 34.55 | 39.40 | 0.367 |
| Clostridia | 5.76 | 14.82 | 0.004* |
| *Veillonella* | 0.71 | 6.61 | 0.007* |
| Bacilli | 28.79 | 23.26 | 0.407 |
| *Staphylococcus* | 19.69 | 8.53 | 0.462 |
| *Streptococcus* | 0.96 | 4.32 | 0.002* |
|  |  |  |  |
|  | **Lethal NEC (n=6)** | **Control (n=21)** | **P-value** |
| **Actinobacteria** | 1.53 | 1.99 | 0.064 |
| Actinobacteria | 1.53 | 1.99 | 0.064 |
| **Proteobacteria** | 65.29 | 59.93 | 0.316 |
| Gammaproteobacteria | 65.30 | 58.63 | 0.351 |
| *Klebsiella* | 6.20 | 3.42 | 0.977 |
| **Firmicutes** | 33.17 | 37.32 | 0.345 |
| Clostridia | 0 | 5.45 | 0.007* |
| *Veillonella* | 0 | 3.30 | 0.047* |
| Bacilli | 33.18 | 31.87 | 0.512 |
| *Staphylococcus* | 32.36 | 13.31 | 0.414 |
| *Streptococcus* | 0.01 | 6.49 | 0.025* |
|  |  |  |  |
|  | **Severe NEC mean** | **Control mean** | **P-value** |
| **Actinobacteria** | 0.17 | 1.74 | 0.034* |
| Actinobacteria | 0.17 | 1.61 | 0.044* |
| **Proteobacteria** | 70.04 | 58.40 | 0.409 |
| Gammaproteobacteria | 68.32 | 57.80 | 0.445 |
| *Klebsiella* | 6.88 | 6.07 | 0.053 |
| **Firmicutes** | 29.14 | 34.93 | 0.566 |
| Clostridia | 3.08 | 11.27 | 0.202 |
| *Veillonella* | 1.85 | 4.06 | 0.419 |
| Bacilli | 26.07 | 23.67 | 0.746 |
| *Staphylococcus* | 21.04 | 11.74 | 0.663 |
| *Streptococcus* | 0.01 | 4.46 | 0.009* |
|  |  |  |  |
|  | **Mild NEC (n=8)** | **Control (n=31)** | **P-value** |
| **Actinobacteria** | 2.08 | 1.49 | 0.900 |
| Actinobacteria | 1.95 | 1.49 | 0.457 |
| **Proteobacteria** | 49.56 | 53.69 | 0.772 |
| Gammaproteobacteria | 49.50 | 53.67 | 0.772 |
| *Klebsiella* | 2.69 | 2.64 | 0.583 |
| **Firmicutes** | 40.30 | 43.98 | 1.000 |
| Clostridia | 12.41 | 23.70 | 0.217 |
| *Veillonella* | 0.25 | 10.67 | 0.061 |
| Bacilli | 27.89 | 17.14 | 0.903 |
| *Staphylococcus* | 9.01 | 3.03 | 0.685 |
| *Streptococcus* | 2.52 | 2.75 | 0.490 |

*Statistically significant at p<0.050.

**Table S3: ANOVA of OTU Abundances:** ANOVA using the OTU abundance table to test whether there were any OTUs that were significantly different between NEC and controls using OTUs present in at least 25% of specimens (102 total OTUs). Four OTUs shown below were found to be significantly different, however, the p-values did not reach significance after False Discovery Rate (FDR) correction. Representative sequences for the OTUs shown are given as well.

| OTU # | Probability | FDR corrected  p-value | Taxonomy |
| --- | --- | --- | --- |
| 78 | 0.002515618 | 0.25659306 | k__Bacteria; p__Proteobacteria; c__Gammaproteobacteria; o__Enterobacteriales; f__Enterobacteriaceae |
| 905 | 0.007121959 | 0.363219904 | k__Bacteria; p__Firmicutes; c__Bacilli; o__Bacillales; f__Staphylococcaceae; g__Staphylococcus; s__epidermidis |
| 916 | 0.010769104 | 0.366149529 | k__Bacteria; p__Firmicutes; c__Bacilli; o__Bacillales; f__Staphylococcaceae; g__Staphylococcus; s__epidermidis |
| 553 | 0.03321927 | 0.847091391 | k__Bacteria; p__Proteobacteria; c__Gammaproteobacteria; o__Enterobacteriales; f__Enterobacteriaceae |

**Representative Sequences from OTUs listed in Table S3:**

OTU 78: CTGGACCGTGTCTCAGTTCCAGTGTGGCTGGTCATCCTCTCAGACCAGCTAGGGATCGTCGCCTAGGTGAGCCGTTACCCCACCTACTAGCTAATCCCATCTGGGCACATCTGATGGCAAGAGGCCCGAAGGTCCCCCTCTTTGGTCTTGCGACGTTATGCGGTATTAGCTACCGTTTCCAGTAGTTATCCCCCTCCATCAGGCAGTTTCCCAGACATTACTCACCCGTCCG

OTU 905: CTGGACCGTGTCTCAGTTCCAGTGTGGCCGATCACCCTCTCAGGTCGGCTACGCATCGTGCCTTGGTAAGCCGTTACCTACCAACTAGCTAATGCGGCGCGGATCCATCTATAAGTGACAGCAAAACCGTCTTTCACTATTGAACCATGCGGTTCAATATATTATCCGGTATTAGCTCCGGTTTCCCGAAGTTATCCCAGTCTTATAGGTAGGTTATCCACGTGTTACTCACCCGTCCG

OTU 916: GATGAACGCTGGCGGCGTGCCTAATACATGCAAGTCGAGCGAACAGACGAGGAGCTTGCTCCTCTGACGTTAGCGGCGGACGGGTGAGTAACACGTGGATAACCTACCTATAAGACTGGGATAACTTCGGGAAACCGGAGCTAATACCGGATAATATATTGAACCGCATGGTTCAATAGTGAAAGACGGGTTTTGCTGTCACTTATAGATGGATCCGCGTCGCATTAGCTAGTTGGTAAGGTAACGGCTTACCAAGGCAACGATGCGTAGCCGACCTGAGAGGGTGATCGGCCACACTGGAACTGAGACAC

OTU 553: ATTGAACGCTGGCGGCAGGCCTAACACATGCAAGTCGAACGGTAGCACAGAGGAGCTTGCTCCTTGGGTGACGAGTGGCGGACGGGTGAGTAATGTCTGGGAAACTGCCCCGATGGAGGGGATAACTACTGGAAACGGTAGCTAATACCGCATAACGTCGCAAGACCAAAGAGGGGACCTTCGGGCCGTCTTGCCATCGGATGTGCCCAGATGGGATTAGCTAGTA

**Figure S1: Diversity of Lethal, Severe, and Mild NEC specimens compared to controls**. Microbial diversity of each severity was measured using Chao1 Richness Estimation and Shannon’s Diversity Index. Lethal NEC (n=6) was compared to 21 matched controls (A,B). Severe NEC (n=7) was compared to 22 matched controls(C,D). Mild NEC (n=8) was compared to 31 controls (E,F). The solid horizontal line represents the mean with standard deviation represented by dashed lines. Significance was measured using Mann-Whitney U-test. *p<0.05; **p<0.01.


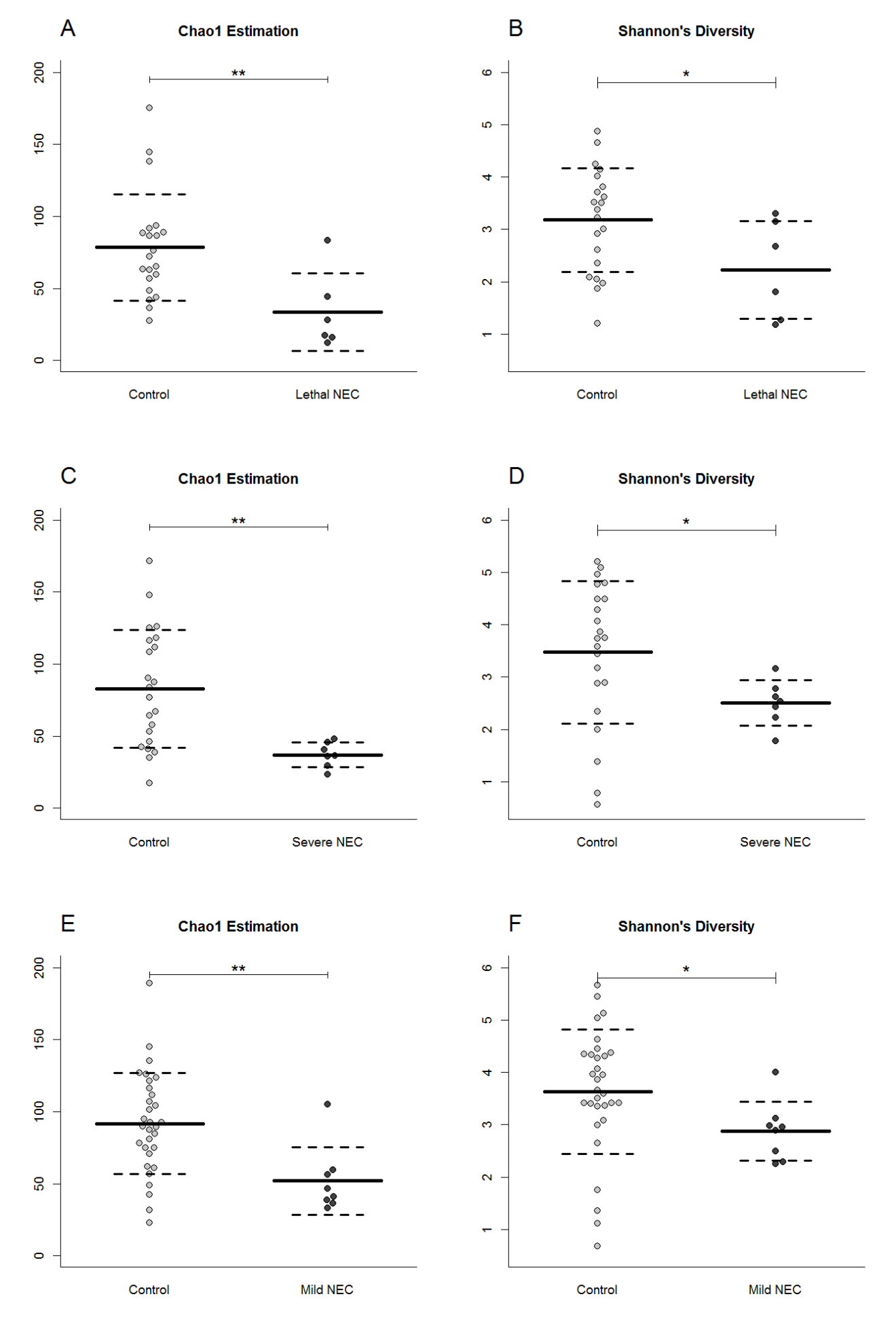


**References:**

1. Olsen IE, Groveman SA, Lawson ML, Clark RH, Zemel BS. New intrauterine growth curves based on United States data. Pediatrics. 2010; 125:e214–24. doi:10.1542/peds.2009-0913.
